# Near-infrared light-activated lidocaine microneedle patch for rapid local anesthesia

**DOI:** 10.7150/thno.129132

**Published:** 2026-03-09

**Authors:** Shuailei Wang, Ze Qiang Zhao, Yumiao He, Bo Zhi Chen, Hongju Liu, Xin Dong Guo, Yuguang Huang

**Affiliations:** 1 Department of Anesthesiology, Peking Union Medical College Hospital, Chinese Academy of Medical Sciences & Peking Union Medical College, Beijing, 100730, China.; 2 State Key Laboratory of Organic-Inorganic Composites, Beijing University of Chemical Technology, Beijing 100029, China.; 3 Beijing Laboratory of Biomedical Materials, College of Materials Science and Engineering, Beijing University of Chemical Technology, Beijing 100029, China.

**Keywords:** lidocaine, local anesthesia, microneedle, pain, MXene

## Abstract

**Rationale:**

Effective local anesthesia with rapid onset and minimal invasiveness is essential for certain minor surgeries, such as aesthetic procedures, but remains difficult to achieve with conventional local anesthesia.

**Methods:**

A photothermal-responsive microneedle system (LiH/MXene@MNs) was developed, consisting of lidocaine-loaded needle tips and a backing layer in which MXene was confined exclusively. This system employs a spatial design that separates the drug and photothermal components, allowing efficient drug delivery under short-term near-infrared (NIR) irradiation. Mechanical, optical, and histological assessments were performed to evaluate the system's properties. *In vitro* and *in vivo* studies were conducted to assess biosafety and invasiveness, while behavioral tests were performed in a rat plantar incision model to evaluate the onset and duration of anesthesia.

**Results:**

The LiH/MXene@MNs system exhibited robust skin penetration and high drug-loading efficiency. Behavioral tests in a rat plantar incision model further verified that anesthesia onset was induced within 5 minutes and sustained for up to 60 minutes on this platform, comparable to that achieved with invasive injection. Additionally, the system exhibited minimal invasiveness and excellent biosafety, as demonstrated by both *in vitro* and *in vivo* studies.

**Conclusions:**

The LiH/MXene@MNs platform offers an efficient, minimally invasive, and safe alternative to traditional local anesthesia techniques. This approach holds strong potential for use in minimally invasive aesthetic procedures, providing a promising solution for rapid and effective pain management

## Introduction

Rapid-onset and minimally invasive local anesthesia is critically needed in a wide range of outpatient and dermatological procedures, including skin biopsies, mole excisions, laser therapy, and minor surgeries. These clinical scenarios often require localized analgesia with minimal systemic side effects and rapid patient throughput 1. However, traditional local anesthesia methods, including injectable and topical formulations, have significant limitations under these conditions. Injected local anesthesia, while fast-acting and effective, requires skilled administration, carries a risk of systemic toxicity 2, and often causes injection-site pain, which can reduce patient compliance. Topical anesthesia, on the other hand, avoids needle-related discomfort but is hindered by the intact stratum corneum barrier, resulting in limited skin penetration, delayed onset, and inconsistent efficacy 3. These challenges underscore the clinical need for a rapid, painless, and convenient local anesthetic delivery platform suitable for pre-procedural application on intact skin.

Nanomedicine and nanotechnology have introduced new possibilities for enhancing local anesthetic delivery 4. For instance, liposomal bupivacaine 5 and bupivacaine-loaded hydrogels 6-8 have been used for postoperative pain management. Yet, these systems generally require injection and primarily focus on sustained-release formulations, and thus lack the capacity to provide rapid-onset local anesthesia. Microneedles (MNs) have emerged as an effective transdermal delivery platform, enabling painless penetration of the stratum corneum, enhanced drug absorption, and improved patient compliance 9. Compared to conventional topical formulations, MNs substantially enhance transdermal delivery efficiency 10. However, conventional MNs rely mainly on passive diffusion of the encapsulated drug, which often results in slow and sometimes inadequate onset of action, thereby limiting their applicability in procedures requiring rapid analgesia 11, 12. To accelerate transdermal transport, MNs and other transdermal systems have therefore been combined with a variety of external-stimulus-based or device-assisted strategies, including iontophoresis 13, ultrasound 14, electroporation 15, and photothermal activation 16. However, iontophoresis-, electroporation-, and ultrasound-assisted drug delivery typically rely on specialized hardware and dedicated treatment sessions, thereby adding extra steps and time to the procedure 17. These electrical, ultrasound-based, or mechanically actuated approaches can be technically demanding and have been associated with skin or mucosal irritation, which may limit their practicality in high-throughput outpatient practice. Moreover, a large proportion of stimulus-responsive MNs and nanoplatforms have been designed for chronic disease management, including vaccination, cancer therapy, and inflammatory disease control 18. These systems generally emphasize sustained or environment-adaptive release rather than on-demand delivery for rapid-onset local analgesia.

Among these strategies, photothermal modulation is attractive because localized mild heating can transiently increase skin permeability on demand, thereby accelerate the onset of local anesthesia without substantially increasing systemic exposure 19. In practice, mild, spatially confined heating can be generated by compact NIR light sources or simple heating elements and is generally well tolerated by patients 20. In particular, light-driven photothermal modulation is highly accessible and compatible with current aesthetic workflows, as light- and laser-based procedures are widely used. As a result, near-infrared (NIR)-induced mild heating is often perceived as a psychologically and cosmetically more acceptable approach for enhancing local anesthetic delivery in these settings. MXenes are a family of emerging two-dimensional transition metal carbides/nitrides with a unique layered structure and a large specific surface area. They exhibit excellent light absorption in the near-infrared (NIR) region and good biocompatibility. As photothermal agents, MXenes efficiently convert NIR light into localized heat, enabling non-invasive, spatiotemporally controlled photothermal modulation that promotes rapid molecular diffusion and on-demand drug release. Compared to traditional photothermal materials, MXenes offer higher photothermal conversion efficiency and a faster thermal response, making them ideal for photothermal therapy and drug delivery systems. This high performance makes MXenes the material of choice for the NIR-responsive drug delivery platform in this study 21, 22. Nevertheless, most existing photothermal delivery systems incorporate the photothermal agent within the drug-loaded region, raising concerns about its direct contact with tissue and making it difficult to optimize heating and drug loading independently. Moreover, reports on integrating microneedles with heat-assisted transdermal strategies that are specifically tailored to optimize the timescale of local anesthesia remain rare.

**Scheme 1:**
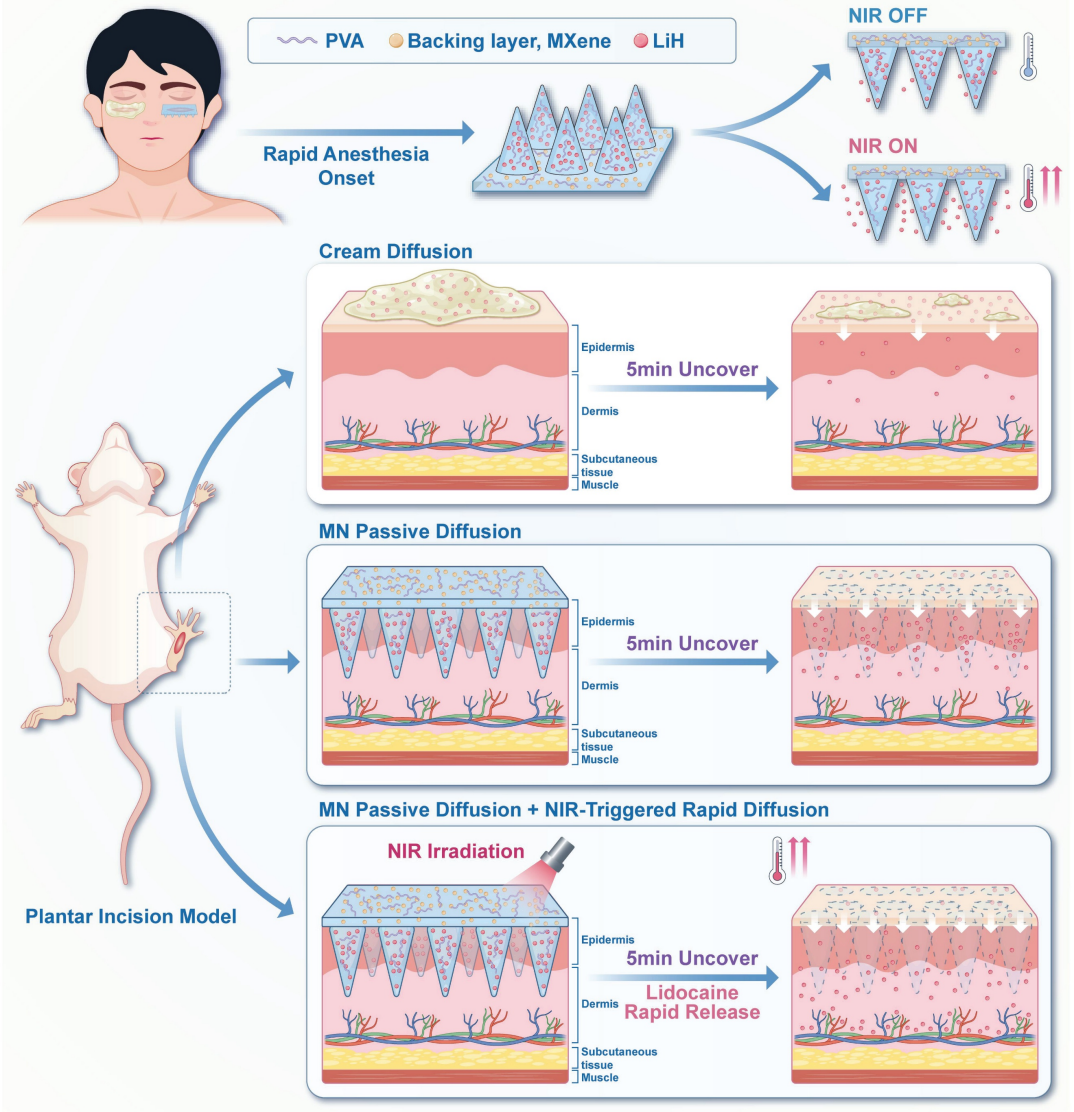
Schematic illustration of LiH/MXene@MNs for rapid-onset local anesthesia. Lidocaine hydrochloride (LiH) is loaded into the microneedles, while MXene is confined to the backing layer. The combination of microneedle-assisted delivery and NIR-induced photothermal heating accelerates the transdermal transport of LiH.

Herein, we propose a photothermal-activated, lidocaine-loaded microneedle system (LiH/MXene@MNs). In this design, lidocaine hydrochloride (LiH), a widely used local anesthetic, is loaded into the needle tips 23, while MXene is exclusively confined to the backing layer (**Scheme 1**). This spatial arrangement prevents MXene from penetrating the skin while enabling precise photothermal control. Under 808 nm NIR irradiation, MXene rapidly generates localized heat, thereby accelerating transdermal diffusion. The microneedle patch is applied locally for just 5 min, ensuring rapid onset and minimizing systemic exposure. Overall, the LiH/MXene@MNs system enhances drug diffusion through photothermal-triggered release, providing a minimally invasive, rapid-onset, highly efficient, and safe solution for local anesthesia in minor procedures.

## Results and Discussion

### Fabrication and characterization of the LiH/MXene@MNs

**Figure 1:**
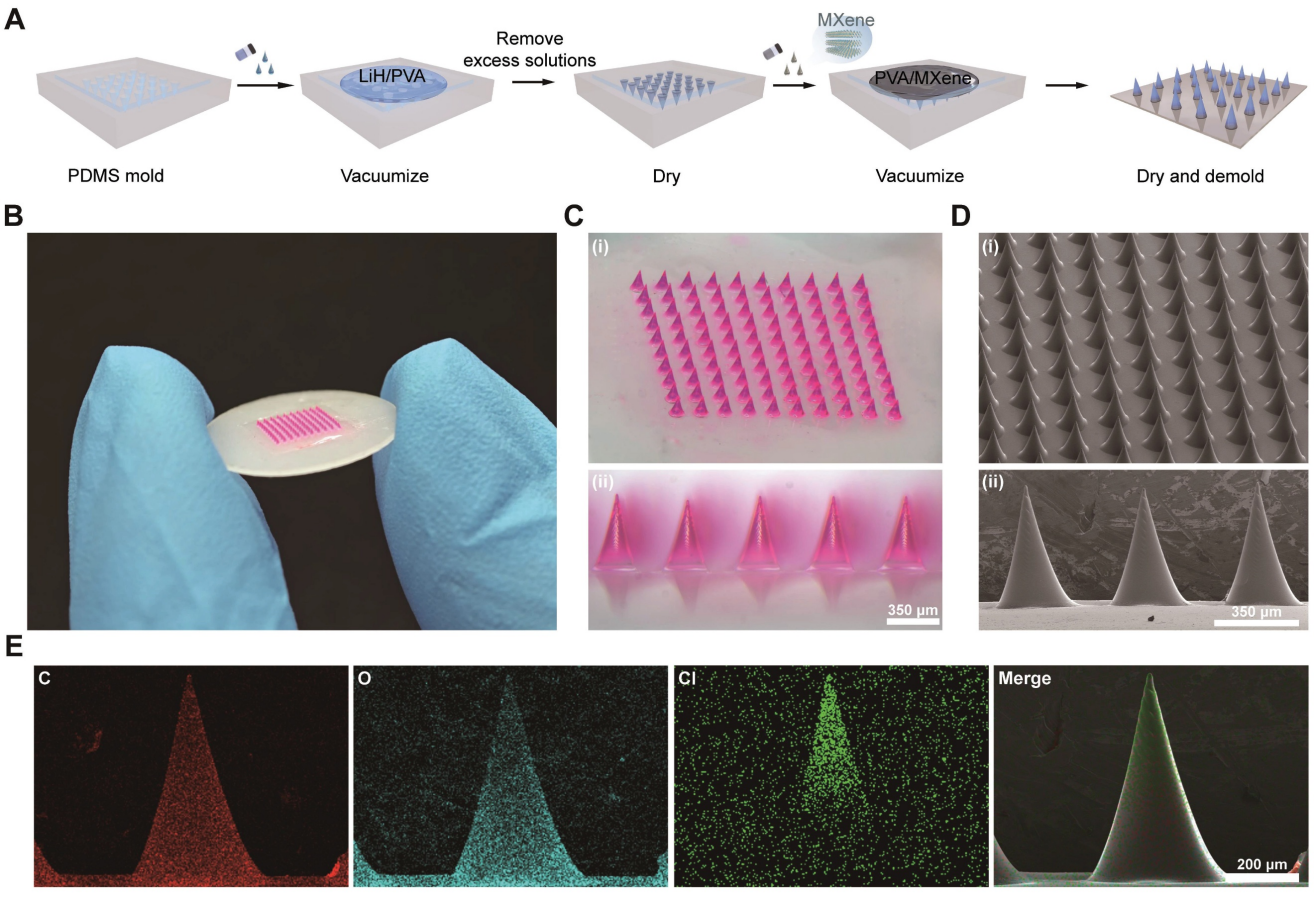
Fabrication process and structural characterization of LiH/MXene@MNs. (A) Schematic illustration of the fabrication process. (B) Photograph of the prepared microneedle (MN) patch. (C) Optical microscopy and (D) Scanning electron microscopy (SEM) image of the MN array at the final selected LiH/PVA ratio (3/4). (E) Elemental mapping of a single MN obtained by energy-dispersive X-ray spectroscopy (EDS). Red represents carbon (C), cyan indicates oxygen (O), and green represents chlorine (Cl).

To meet the clinical need for rapid-onset local anesthesia, we designed LiH/MXene@MNs by combining photothermal-responsive MXene in the patch backing with lidocaine-loaded polyvinyl alcohol (PVA) microneedle tips. PVA, chosen for its water solubility, biocompatibility, and low cost 24, 25, was selected as the scaffold material for the microneedle matrix. Sucrose was added to enhance hydrogen bonding and mechanical strength 26. To prevent penetration of the photothermal agent into tissue, MXene was confined exclusively to the backing layer. This compartmentalized structure ensures biological safety while maintains drug delivery efficiency. The fabrication process involved two steps: (1) loading LiH/PVA into a polydimethylsiloxane (PDMS) mold under vacuum to form MNs, and (2) adding an MXene/PVA solution to the mold to form the backing layer. After vacuum drying, a structurally robust, NIR-responsive microneedle patch was successfully obtained (**Figure 1A**).

To facilitate visualization, rhodamine B was incorporated into the microneedle matrix. Optical microscopy revealed that the prepared MNs were micron-sized, with an overall patch size approximately similar to that of a coin (**Figure 1B**). A complete 10 × 10 MN array was obtained, with all tips remaining intact (**Figure 1C**). The needles showed a conical geometry, reaching approximately 600 μm in height with a basal diameter of 350 μm (**Figure 1D**). Scanning electron microscopy (SEM) revealed smooth microneedle surfaces without crystallization, indicating homogeneous dispersion of the drug. Energy-dispersive X-ray spectroscopy (EDS) mapping showed that Cl (from LiH) was concentrated in the upper half of the needle, optimizing the drug delivery depth (**Figure 1E**), while Ti (from MXene) was almost entirely localized in the backing layer and partially in the basal region of the MNs (**Figure S1**).

### Loading efficiency and mechanical properties of MN patches

**Figure 2:**
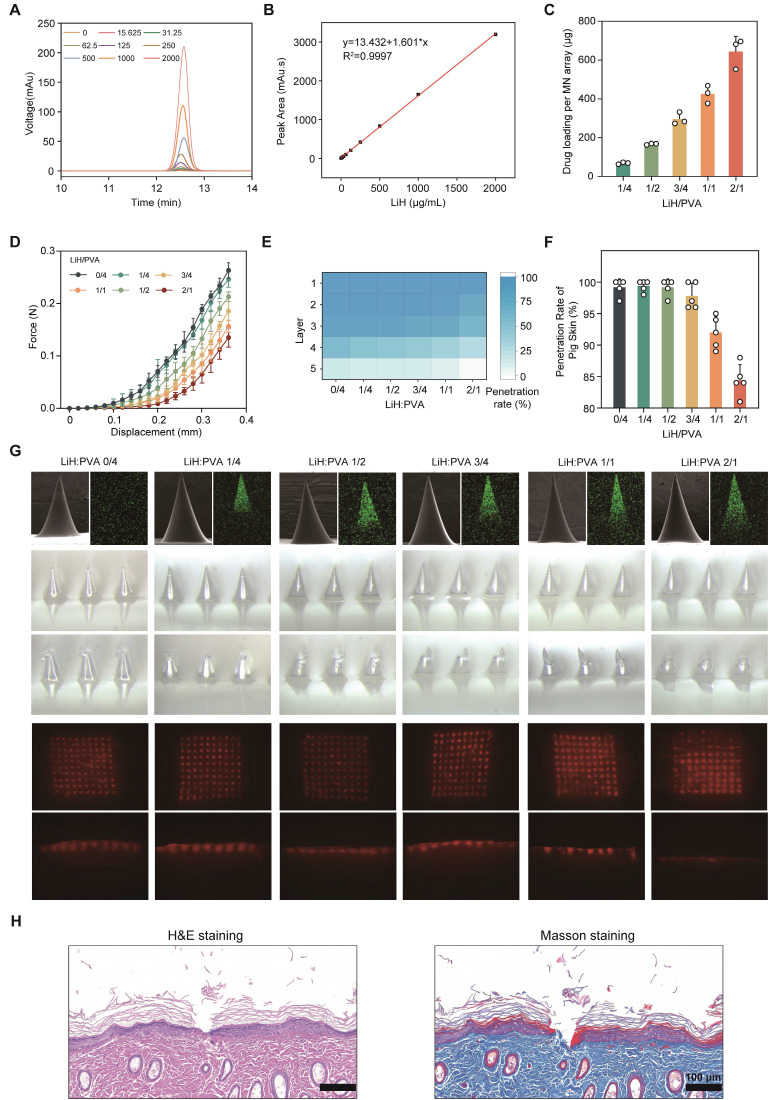
Drug loading and mechanical properties of LiH/MXene@MNs. (A) HPLC chromatograms of LiH at different concentrations. (B) Standard curve of LiH established. (C) Drug loading of the MN patch at different LiH/PVA ratios (n = 3). (D) Force-displacement curves at different LiH/PVA ratios (n = 5). (E) Number of penetrated layers in a Parafilm simulated membrane at different LiH/PVA ratios (n = 3). (F) Penetration rate in porcine skin at different LiH/PVA ratios (n = 5). (G) MN morphology and penetration visualization at different LiH/PVA ratios (n = 3). Note: The representative morphology image for LiH/PVA = 3/4 shown in panel G is the same source image as that shown in Figure 1D, as both depict the final selected formulation (LiH/PVA = 3/4). (H) H&E and Masson's staining images of MN insertion (n = 3). Scale bar: 100μm. All data are shown as mean ± standard error (SE).

To quantify the LiH content in each MN patch with varying LiH concentrations, high-performance liquid chromatography (HPLC) was performed on the dissolved patches, and drug loading was calculated from the peak areas (**Figure 2A,B**). Regardless of the mechanical properties, successful microneedle formation was achieved at the LiH/PVA mass ratios of 1/4, 1/2, 3/4, 1/1, and 2/1, with the corresponding LiH loadings in LiH/MXene@MNs of 69.00 ± 4.98, 167.49 ± 3.87, 296.52 ± 31.53, 425.35 ± 45.88, and 643.57 ± 79.05 µg per MN patch (per MN array), which were sufficient to meet the local anesthesia requirements (**Figure 2C**).

To balance drug content with structural integrity, we investigated the relationship between LiH/PVA ratios and mechanical strength 27. As the LiH content increased, the mechanical performance of the MNs decreased markedly (**Figure 2D**). In the simulated skin penetration test using a Parafilm sealing membrane, MNs with LiH/PVA ratios from 0/4 to 3/4 were able to pierce all three layers of the membrane, whereas the penetration rate decreased substantially at 1/1 and 2/1 (**Figure 2E**). Experiments on porcine skin confirmed these findings via fluorescence imaging and histological sections, showing reduced penetration depth in MNs with higher drug content (**Figure 2F,G**). This effect is attributed to weakened polymer network integrity: PVA provides mechanical strength through its chain structure, whereas excessive LiH lacks sufficient intermolecular bonding, resulting in mechanical failure under stress 28. Based on these findings, a LiH/PVA mass ratio of 3/4 was selected as the optimal ratio, offering high drug loading without compromising mechanical performance. H&E and Masson staining demonstrated that these MNs effectively penetrated rat dorsal and plantar skin (**Figure 2H and Figure S2**).

### NIR-activated photothermal effect of LiH/MXene@MNs *in vivo*

**Figure 3:**
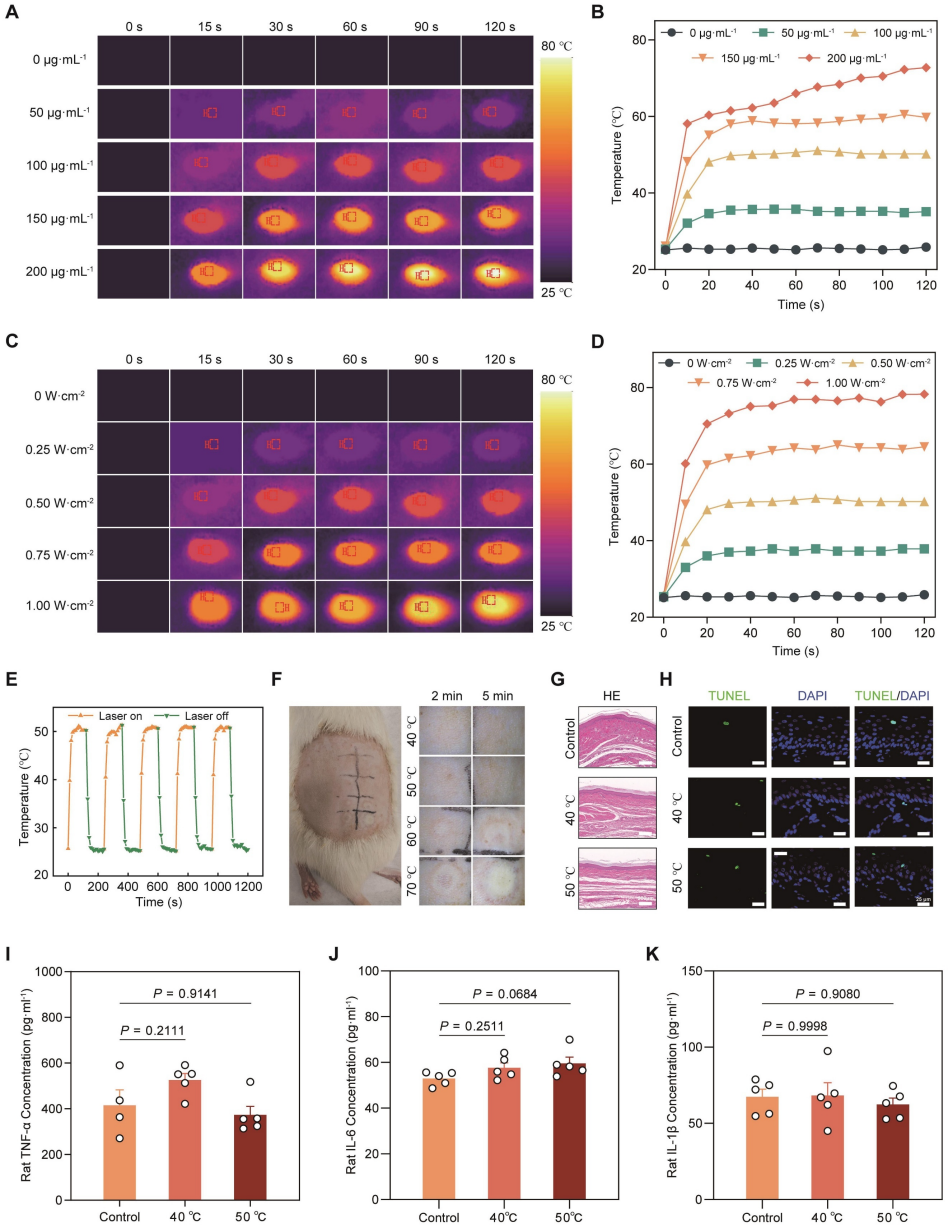
*In vivo* photothermal effect and local biosafety evaluation of LiH/MXene@MNs. (A) Thermal images and (B) temperature-time curves of LiH/MXene@MNs at different MXene concentrations under 808 nm NIR irradiation (0.5 W·cm⁻²). (C) Thermal images and (D) temperature-time curves of LiH/MXene@MNs under different power densities (MXene 100 μg·mL⁻¹; 808 nm NIR). (E) Transient thermal response of LiH/MXene@MNs (MXene 100 μg·mL⁻¹) over five on/off irradiation cycles (0.5 0.5 W·cm⁻², 808 nm NIR). (F) Photographs of rat dorsal skin treated with LiH/MXene@MNs at different target temperatures (40, 50, 60, and 70 °C) for 2 and 5 min under 808 nm NIR irradiation (n = 3). (G-H) H&E and TUNEL staining of rat plantar skin after LiH/MXene@MNs treatment with/without 808 nm NIR irradiation, with the local skin surface temperature maintained at 40 or 50 °C for 2 min (n = 3). Scale bars: 200 µm (H&E) and 25 µm (TUNEL). (I-K) ELISA quantification of inflammatory cytokines (TNF-α, IL-6, and IL-1β) in rat plantar skin tissues after LiH/MXene@MNs treatment with/without 808 nm NIR irradiation (40 or 50 °C for 2 min; n = 5).

To evaluate the thermal responsiveness of MXene in the MN patch, we systematically tested its photothermal properties at varying MXene concentrations (0, 50, 100, 150, 200 μg·mL⁻¹) and irradiation powers (0, 0.25, 0.5, 0.75, 1 W·cm⁻²) in rats *in vivo*. The results indicated that as the MXene concentration increased, the temperature-rise capacity of the MN patch gradually improved. Under 808 nm laser irradiation, LiH/MXene@MNs displayed a concentration-dependent temperature elevation, with higher MXene loadings producing markedly greater heat generation (**Figure 3A,B**). In addition, the temperature rise showed a clear power-dependent trend, with higher laser power resulting in greater temperature elevations, and no obvious inflection points were observed (**Figure 3C,D**). At 100 μg·mL⁻¹ MXene and an irradiation power density of 0.5 W·cm⁻², the patch reached approximately 50°C within 2 min in rats *in vivo*, ensuring rapid diffusion. Furthermore, after five cycles of testing, the MN patch maintained a consistent temperature increase during photothermal activation, with no significant performance degradation (**Figure 3E**). These results further confirm that MXene not only possesses excellent photothermal properties in the MN system but also demonstrates good thermal stability, indicating its potential for long-term use and repeated irradiation in practical applications.

To assess the safety of photothermal heating, we further investigated the effects of different temperatures and treatment durations on skin tissue. Considering patient tolerance and clinical acceptability, we selected 2 and 5 min as the appropriate photothermal treatment durations. The results indicated that at 40 ℃ and 50 ℃, no noticeable damage was observed in rat dorsal skin. However, when the temperature increased further, white or yellow swelling appeared, along with burns and inflammatory reactions (**Figure 3F**). To further evaluate local biosafety under conditions relevant to plantar application, we applied LiH/MXene@MNs to rat plantar skin and performed histological and inflammatory assessments after NIR activation with the local surface temperature maintained at 40 °C or 50 °C for 2 min. H&E staining revealed no evident tissue injury or morphological abnormalities (**Figure 3G**). TUNEL staining showed no obvious increase in apoptosis in either group (**Figure 3H**). In parallel, ELISA analysis of TNF-α, IL-6, and IL-1β in plantar skin homogenates showed comparable cytokine levels between the 40 °C and 50 °C groups, indicating no significant difference in inflammation (**Figure 3I-K**). Based on these findings, 50 °C was selected as the optimal photothermal condition for subsequent permeation experiments (MXene 100 μg·mL⁻¹; irradiation power density 0.5 W·cm⁻²).

### Release kinetics of LiH from LiH/MXene@MNs *in vitro* and *in vivo*

**Figure 4:**
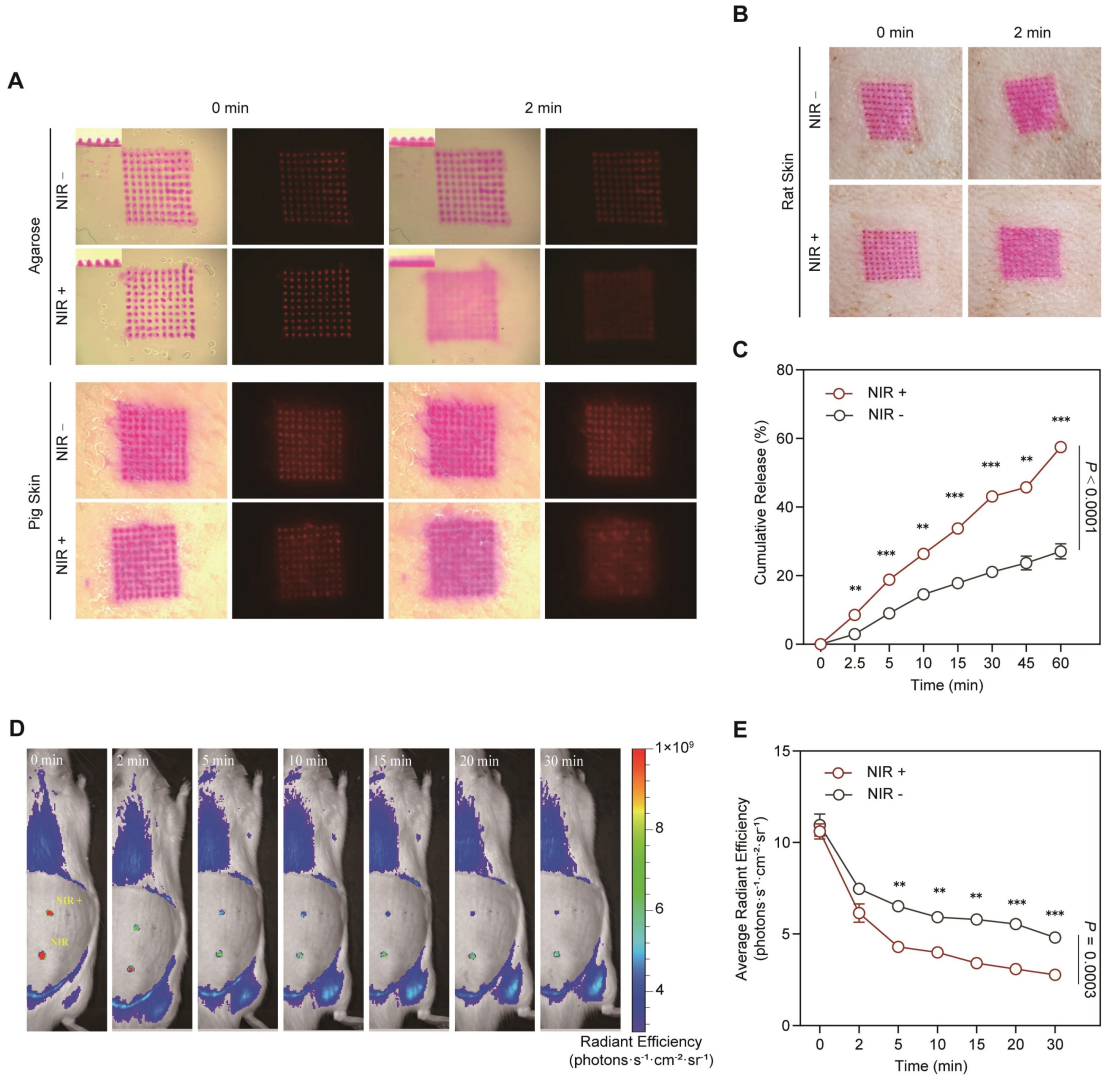
Drug release and absorption properties of LiH/MXene@MNs. (A) *In vitro* simulated drug release and absorption in agarose gelatin and porcine skin. (B) *In vivo* simulated drug release and absorption in rat dorsal skin. (C) Cumulative drug release from LiH/MXene@MNs measured using a Franz diffusion cell (n = 3). (D) Cy3-labeled drug diffusion images and (E) average radiant efficiency in rat skin at multiple time points (0-30 min) under NIR (+) and NIR (-) conditions *in vivo* (n = 3). All data are shown as mean ± SE (^*^ *P* < 0.05, ^**^ *P* < 0.01, and ^***^ *P* < 0.001).

To investigate the impact of NIR-induced photothermal effects on transdermal drug delivery, the prepared MNs were subjected to both *in vitro* and *in vivo* drug release studies. First, LiH/MXene@MNs were inserted into *in vitro* simulated skin models, including agarose-gelatin and porcine skin. Upon NIR irradiation, the patch temperature increased to 50°C and was maintained for two min, significantly enhancing the diffusion of the model drug pigment from the patch (**Figure 4A**). Furthermore, the MN patch was applied to the dorsal skin of rats, and the results were consistent with those of the *in vitro* experiments (**Figure 4B**). NIR irradiation significantly promoted drug diffusion (**Figure 4A,B**). To quantitatively analyze these findings, a Franz diffusion cell was used to evaluate the *in vitro* transdermal diffusion of LiH. The results showed that the cumulative release rate was 19% at 5 min, and 60% at 60 min (**Figure 4C**). Furthermore, under NIR irradiation, the cumulative release rates at all measurement points were significantly higher than those under the non-irradiated condition. Additionally, an MN patch loaded with Cy3 fluorescent dye was applied to the dorsal skin of rats, and *in vivo* imaging revealed that NIR activation significantly accelerated *in vivo* absorption of the drug at multiple time points (5 min, 10 min, 15 min, 20 min, 30 min) (**Figure 4D,E**). These results demonstrate that photothermal activation accelerates transdermal delivery, enabling the rapid attainment of therapeutic drug levels at the local site.

### Biosafety assessment *in vitro*

**Figure 5:**
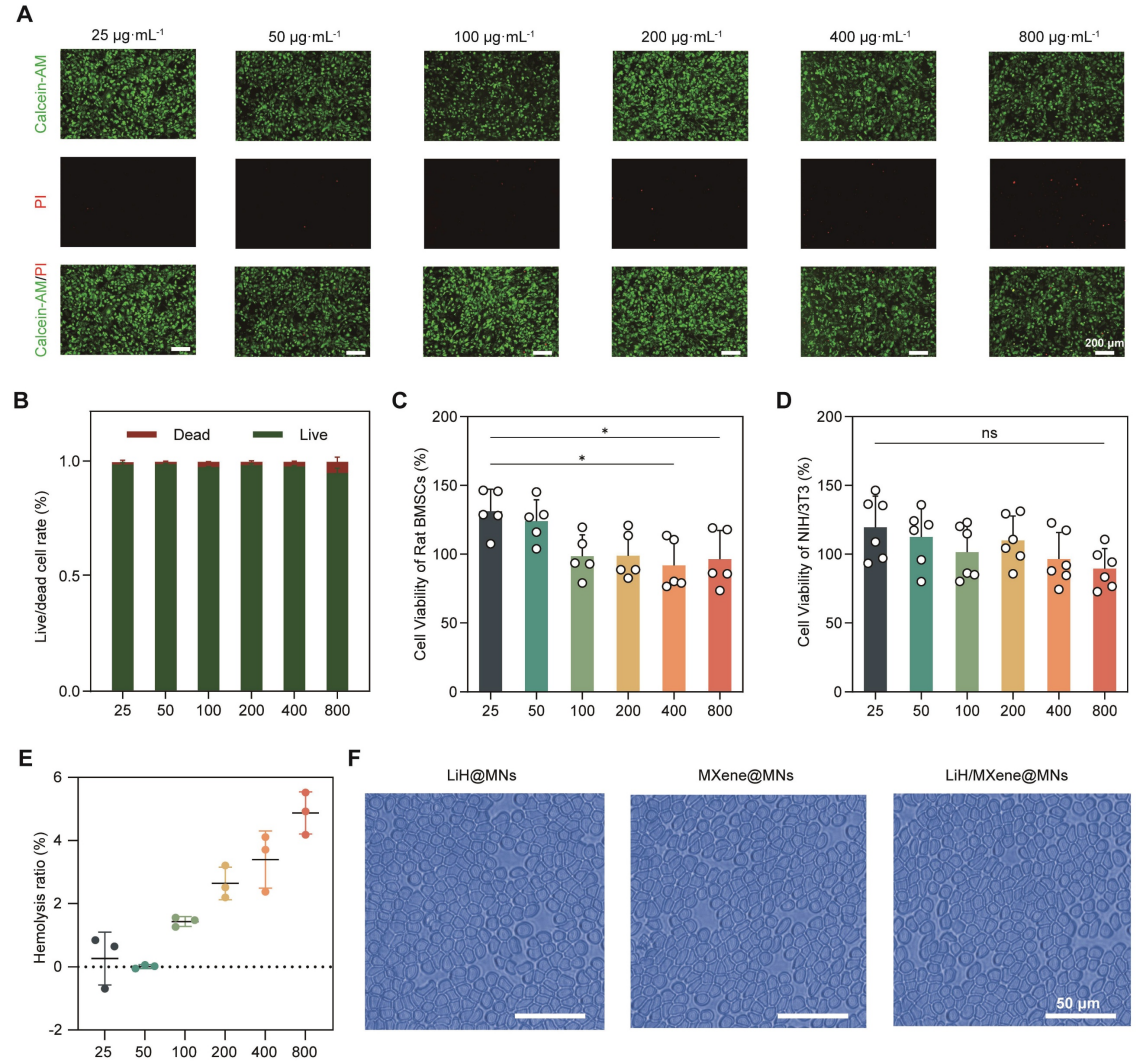
Biosafety assessment of LiH/MXene@MNs *in vitro*. (A) Calcein-AM/PI staining and (B) live/dead cell ratio of BMSCs incubated with different concentrations of LiH/MXene@MNs (n = 3). Scale bar: 200μm. Cell viability of (C) rat BMSCs and (D) mouse NIH/3T3 incubated with different concentrations of LiH/MXene@MNs as measured by the CCK-8 assay. (E) Hemolysis rate of red blood cells incubated with different concentrations of LiH/MXene@MNs (n = 3). (F) Hemolysis rate of red blood cells incubated with LiH@MNs, MXene@MNs and LiH/MXene@MNs (800 μg·mL⁻¹). Scale bar: 50μm. All data are shown as mean ± SE (ns: not significant and ^*^ *P* < 0.05).

Lidocaine achieves local anesthesia and analgesia by blocking nerve endings in the surgical area. However, the risk of substantial systemic absorption via blood vessels and potential material toxicity in clinical applications necessitate comprehensive biosafety evaluations prior to animal studies 29. At the cellular level, Calcein-AM/PI double staining was used to evaluate the cytotoxicity of LiH/MXene@MNs toward bone marrow derived mesenchymal stem cells (BMSCs) 30. The results showed that after treatment with 25-800 μg·mL⁻¹ LiH/MXene@MNs, the majority of BMSCs remained viable (**Figure 5A**). ImageJ analysis showed that the proportion of live cells exceeded 95%, and only a small number of PI-positive cells were observed (**Figure 5B**). The CCK-8 assay results further demonstrated that at concentrations of 400 and 800 μg·mL⁻¹, BMSCs viability slightly decreased but remained above 92% (**Figure 5C**). NIH/3T3 cell viability showed no significant differences across all groups (**Figure 5D**), indicating that LiH/MXene@MNs exhibited good cellular compatibility 31. In terms of hemocompatibility, according to ISO 10993-5 32, medical devices with a hemolytic ratio of <5% are classified as non-hemolytic. The results indicated that low concentrations caused negligible hemolysis, with a slight increase at higher concentrations. Even at 800 μg·mL⁻¹, the hemolysis rate remained below the 5% safety threshold (**Figure 5E**), and the morphology of red blood cells remained intact, with no significant structural damage (**Figure 5F and Figure S3**). Overall, LiH/MXene@MNs exhibited good cellular and hemocompatibility across a wide range of concentrations, suggesting that it maintains a favorable safety profile while ensuring local anesthetic activity.

### LiH/MXene@MNs enables rapid-onset local anesthesia

**Figure 6:**
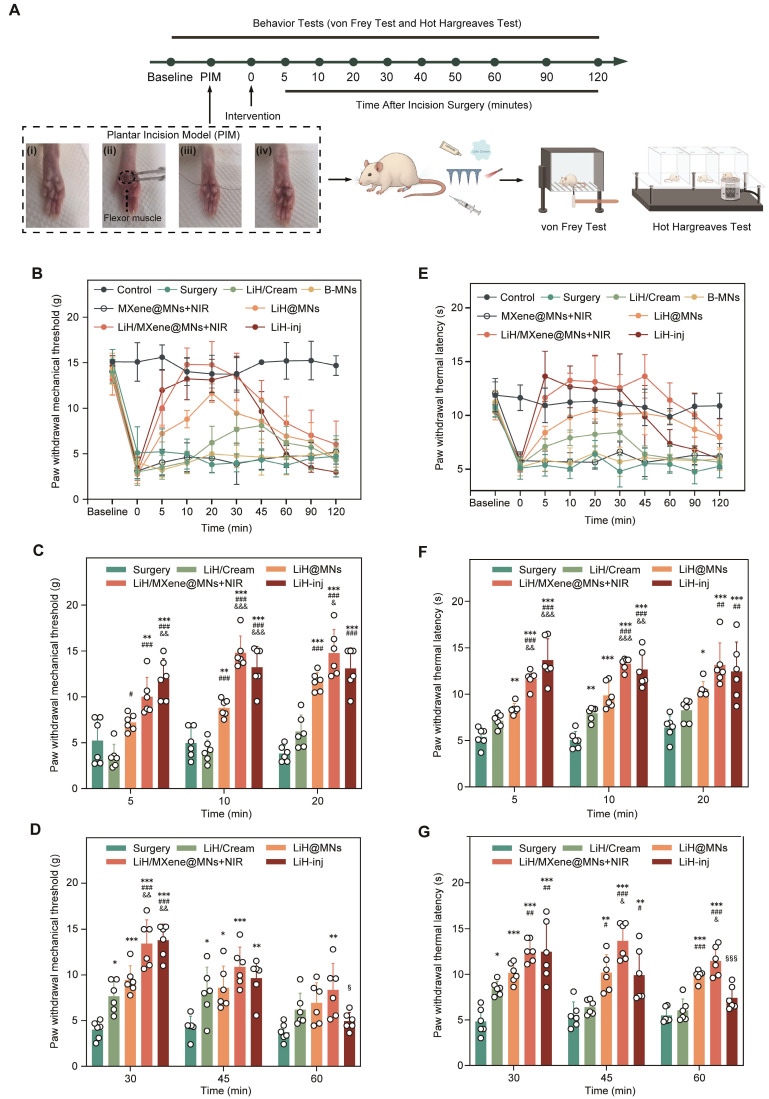
Behavioral tests for rapid anesthetic assessment. (A) Timeline, plantar incision model, and flowchart of behavioral tests. Group definitions: Control, non-incised rats; Surgery, PIM without treatment; B-MNs, blank microneedles (no drug); MXene@MNs + NIR, microneedles with an MXene backing layer only (no lidocaine) under NIR irradiation (heat-only control); LiH/Cream, topical lidocaine cream; LiH@MNs, passive lidocaine-loaded microneedles (no NIR); LiH/MXene@MNs + NIR, lidocaine-loaded, MXene-backed microneedles under NIR irradiation; LiH-inj, local lidocaine injection. (B) Figurative summary of paw withdrawal mechanical threshold over time across different treatment groups. (C-D) Analysis of paw withdrawal mechanical threshold at different time points in Surgery, LiH/Cream, LiH@MNs, LiH/MXene@MNs + NIR and LiH-inj groups (n = 6). (E) Figurative summary of paw withdrawal thermal latency over time across different groups. (F-G) Analysis of paw withdrawal thermal latency at different time points in Surgery, LiH/Cream, LiH@MNs, LiH/MXene@MNs + NIR and LiH-inj groups (n = 6). Statistical symbols apply to panels C, D, F and G. ^*^ *P* < 0.05, ^**^ *P* < 0.01, ^***^ *P* < 0.001 vs. Surgery; ^#^ *P* < 0.05, ^##^ *P* < 0.01, ^###^ *P* < 0.001 vs. LiH/Cream; ^&^ *P* < 0.05, ^&&^ *P* < 0.01, ^&&&^ *P* < 0.001 vs. LiH@MNs; ^§^ *P* < 0.05, ^§§^ *P* < 0.01, ^§§§^ *P* < 0.001 vs. LiH-inj.

To validate the rapid anesthetic performance of the LiH/MXene@MNs system in a clinically relevant context, we used a well-established plantar incision model (PIM) in rats, which closely mimics postoperative pain conditions. At 36 h after the operation, different treatment modalities were applied to the injured hind paw, including blank microneedles (B-MNs), lidocaine cream (LiH/Cream), passive lidocaine-loaded microneedles (LiH@MNs), MXene-only microneedles (MXene@MNs; MXene backing layer without lidocaine) with NIR irradiation (MXene@MNs + NIR), NIR-activated lidocaine-loaded photothermal microneedles (LiH/MXene@MNs + NIR), and local lidocaine injection (LiH-inj). Behavioral assessments were conducted using von Frey filaments for mechanical pain and the Hargreaves test for thermal pain at specific time points (**Figure 6A**). To exclude the potential analgesic contribution from photothermal heating alone, MXene@MNs + NIR was used as a heat-only control under identical irradiation conditions. All groups were anesthetized with 2% isoflurane for 5 min during treatment, and the rats recovered within 5 min without affecting subsequent behavioral tests. In the von Frey test, the surgery group exhibited a pronounced decrease in paw withdrawal threshold (PWT), reflecting mechanical hypersensitivity. The B-MNs(no drug) failed to alleviate pain, confirming the absence of analgesic effects from the microneedle structure and its constituent materials. The LiH/Cream group showed a modest increase in PWT at 30 min post-treatment, with effects lasting approximately 15 min (**Figure 6B**). LiH@MNs produced a faster onset (10 min) and a longer duration of analgesia (30 min), supporting enhanced transdermal delivery via microneedles. Notably, the heat-only control (MXene@MNs + NIR) did not improve PWT relative to the Surgery or B-MNs groups, indicating that NIR-induced heating alone does not account for analgesia. By comparison, LiH/MXene@MNs under NIR irradiation achieved a significantly faster onset (as early as 5 min) and sustained analgesic effects for up to 60 min, outperforming all other non-injected groups (**Figure 6C, D**). This performance was comparable to the LiH-inj group, which began to relieve pain at 5 min and lasted for approximately 45 min. Importantly, LiH/MXene@MNs + NIR group restored near-baseline PWT values within 30 min, indicating robust and rapid suppression of mechanical allodynia. In the Hargreaves thermal test, similar trends were observed (**Figure 6E**).

Consistently, the heat-only control (MXene@MNs + NIR) also showed no improvement in PWL compared with the surgery or B-MNs groups (**Figure 6E**). The LiH/MXene@MNs + NIR group exhibited a significantly faster onset and prolonged paw withdrawal latency (PWL) compared with the LiH/Cream and LiH@MNs groups (**Figure 6F-G**). The analgesic effect persisted up to 60 min, again approaching the efficacy achieved by lidocaine injection. These findings collectively demonstrate that NIR-activated photothermal MNs can rapidly enhance lidocaine transdermal delivery, resulting in quick and sustained analgesia, while avoiding the invasiveness or systemic exposure risks associated with injection. These findings highlight the promising potential of this platform for perioperative pain management in minimally invasive clinical scenarios.

**Figure 7:**
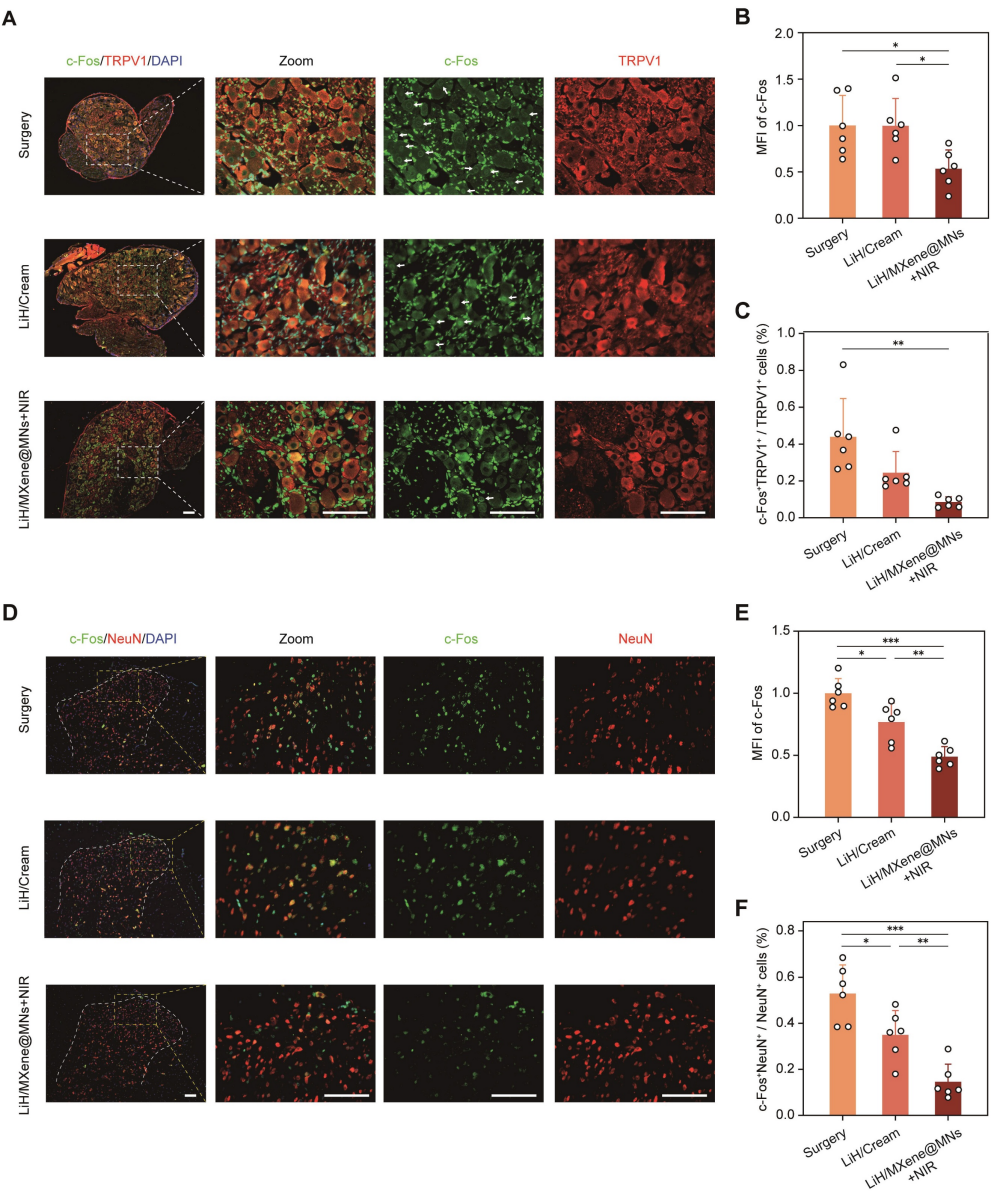
Immunofluorescence staining of c-Fos/TRPV1 and c-Fos/NeuN at 1 h post-treatment. Group definitions: Surgery, PIM without treatment; LiH/Cream, topical lidocaine cream; LiH/MXene@MNs + NIR, lidocaine-loaded, MXene-backed microneedles under NIR irradiation. (A) Representative immunofluorescence images of c-Fos (green) and TRPV1 (red) staining in L4-L5 DRG. DAPI (blue) was used to stain the nuclei. (B) Mean fluorescence intensity (MFI) of c-Fos in different groups. (C) Proportion of TRPV1⁺ neurons that were c-Fos positive in each group. (D) Representative immunofluorescent images of c-Fos (green) and NeuN (red) staining in the SG region of the spinal dorsal horn. DAPI (blue) labels nuclei. (E) c-Fos mean fluorescence intensity in each group. (F) Proportion of NeuN⁺ neurons that were c-Fos⁺ in different groups. All data are shown as mean ± SE (n = 6 slides/group from 6 rats) and analyzed by one-way ANOVA (^*^ *P* < 0.05, ^**^ *P* < 0.01, and ^***^ *P* < 0.001). Scale bars:100 μm.

To provide supportive, functional confirmation of the rapid-onset anesthesia observed above, we assessed neuronal activation markers at both peripheral and spinal levels (**Figure 7**). Primary sensory neurons in the dorsal root ganglion (DRG) primarily mediate peripheral nociceptive signaling 33, and tissue injury induces rapid neuronal activation that can be reflected by upregulated c-Fos expression in both the DRG and spinal dorsal horn 29, 34, 35. Accordingly, c-Fos is commonly used as a marker of nociceptive neuronal activation in the DRG and the substantia gelatinosa (SG) region of the spinal dorsal horn. Additionally, transient receptor potential vanilloid 1 (TRPV1), a capsaicin- and heat-sensitive ion channel, is predominantly expressed in a specific subset of nociceptive neurons, where its upregulation and sensitization under inflammatory conditions contribute to peripheral hyperalgesia 36. Based on the behavioral onset profile, rats received different treatments 36 h after surgery, and lumbar DRG (L4-L5) and dorsal horn tissues were collected 1 h post-treatment for immunofluorescence analysis. First, immunofluorescence staining of L4-L5 DRG sections revealed a marked increase in c-Fos and TRPV1-coexpressing neurons in the surgery group, indicating significant neuronal activation (**Figure 7A**). Compared to the surgery group, the LiH/Cream group only exhibited a reduction in the mean fluorescence intensity (MFI) of c-Fos, while the proportion of c-Fos^+^ neurons among TRPV1 neurons remained unchanged. In contrast, the LiH/MXene@MNs + NIR group exhibited significant reductions in both the MFI of c-Fos^+^ neurons and the proportion of c-Fos^+^ neurons among TRPV1 neurons (**Figure 7B, C**). We acknowledge that while thermal activation may have played a role, the primary effect observed in the LiH/MXene@MNs + NIR group is likely due to the pharmacological action of lidocaine, which is enhanced by the photothermal effect. The temperature increase induced by NIR irradiation returned to baseline within approximately 3 min, suggesting that the observed neuronal inhibition is primarily attributed to lidocaine's action facilitated by heating, rather than heat alone (**Figure S4**). These results support effective peripheral target engagement and are consistent with rapid suppression of nociceptive neuronal activation following LiH delivery by LiH/MXene@MNs under NIR irradiation.

At the spinal cord level, we examined the SG in the dorsal horn, which is the primary synaptic site of DRG neurons. Neuronal activation in this region is also reflected by c-Fos expression in NeuN⁺ spinal neurons 29, 34. Dual fluorescent immunostaining for c-Fos and neuronal nuclei (NeuN) showed extensive neuronal activation in the surgery group, while the LiH/MXene@MNs + NIR group significantly downregulated both c-Fos intensity and the proportion of c-Fos⁺ neurons within the NeuN⁺ population (**Figure 7D-F**). Notably, the suppression effect observed in the LiH/MXene@MNs + NIR group was more pronounced than that of the LiH/Cream group. Together, the DRG and spinal dorsal horn data provide supportive, functional confirmation that LiH/MXene@MNs rapidly attenuates nociceptive neuronal activation *in vivo* under NIR irradiation, in agreement with the rapid-onset behavioral outcomes. Spinal dorsal horn glial markers (Iba-1 and GFAP) were additionally assessed and are presented in the supplementary file (**Figure S5**).

### Biosafety assessment *in vivo*

**Figure 8:**
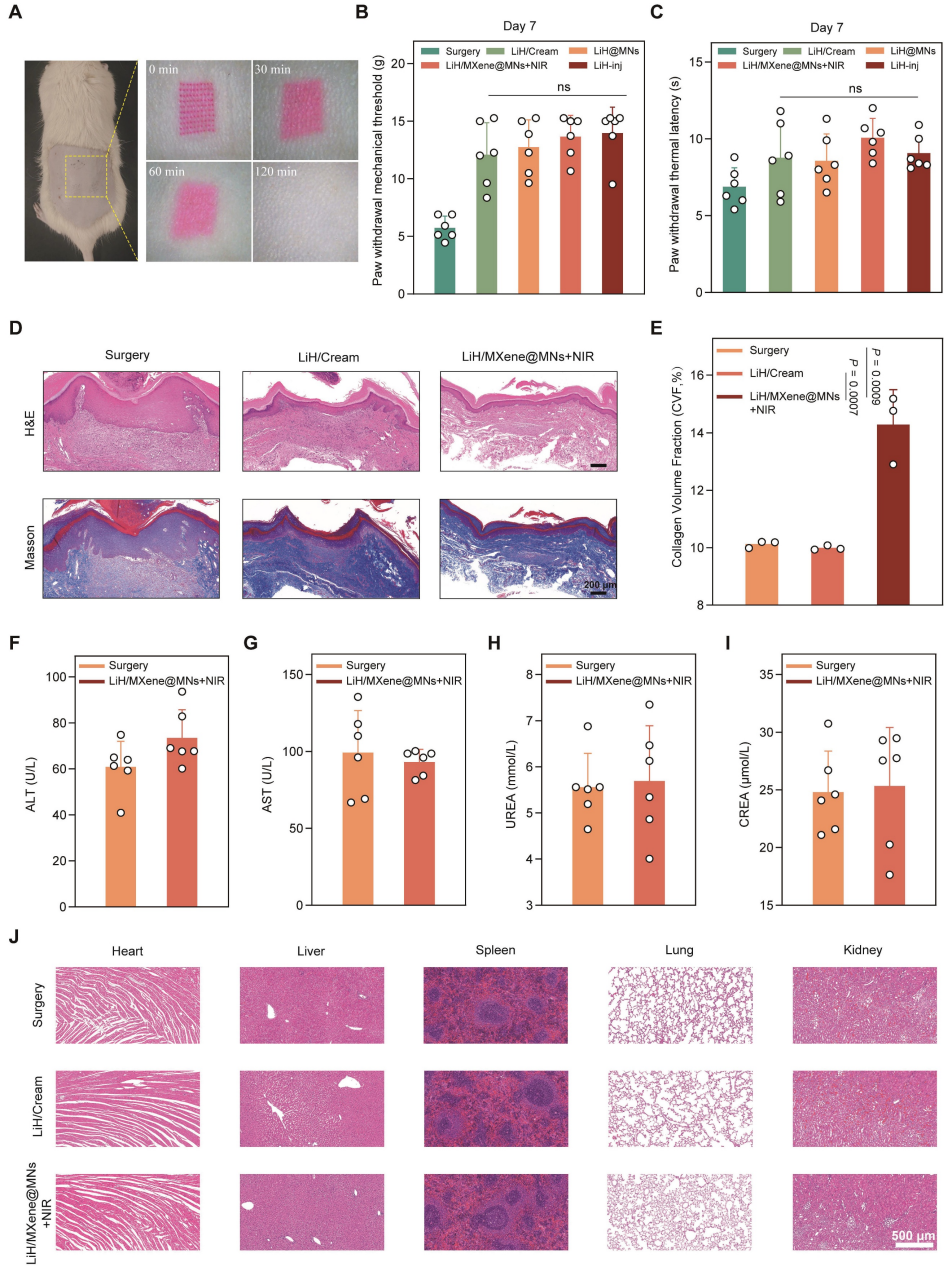
Wound healing and biosafety assessment *in vivo*. Group definitions: Surgery, PIM without treatment; LiH/Cream, topical lidocaine cream; LiH@MNs, passive lidocaine-loaded microneedles (no NIR); LiH/MXene@MNs + NIR, lidocaine-loaded MXene-backed microneedles under NIR irradiation; LiH-inj, local lidocaine injection. (A) Representative images of insertion-site recovery after MN application at 2 h. (B) Paw withdrawal mechanical threshold 7 days post-surgery in different groups. (C) Paw withdrawal thermal latency 7 days post-surgery in different groups. (D) H&E and Masson's staining of the incisional tissues 7 days post-surgery in different groups. Scale bars: 200 μm. (E) Quantitative analysis of collagen volume fraction based on Masson's staining (n = 3). (F-I) The values of ALT, AST, UREA and CREA in rats 1 week after different treatments (n = 6). (J) H&E staining of the heart, liver, spleen, lung, kidney in rats in different groups 1 week post-treatment. Scale bars: 500 μm. All data are shown as mean ± SE (ns: not significant, ^*^ *P* < 0.05, ^**^ *P* < 0.01, and ^***^ *P* < 0.001).

Ensuring tissue compatibility and minimizing interference with the natural healing process are critical for any transdermal delivery platform. First, rhodamine B-labeled MNs were applied to the dorsal skin of rats to visually assess insertion-site recovery. The results showed that puncture marks were nearly resolved within 2 h post-treatment, with no evidence of edema, erythema, or infection, highlighting the minimally invasive nature of the patch (**Figure 8A**). Next, pain-related behaviors were evaluated 7 days post-surgery to assess the short-term impact of the treatment. No significant differences were observed in PWT or PWL across all groups, suggesting that MN treatment exerted no adverse influence on the recovery trajectory (**Figure 8B,C**). However, histological analysis revealed distinct differences in wound healing among the groups. H&E staining demonstrated organized epidermal and dermal structures in the LiH/MXene@MNs + NIR group, with markedly less inflammatory infiltration compared with the LiH/Cream and surgery groups (**Figure 8D**). Masson's staining revealed dense and orderly collagen deposition in the LiH/MXene@MNs + NIR group's wounds, in contrast to the disorganized and sparse collagen observed in the LiH/Cream group. Quantification of collagen volume fraction (CVF) confirmed that the LiH/MXene@MNs + NIR group achieved the highest CVF among all groups, indicating enhanced extracellular matrix remodeling and tissue regeneration 29 (**Figure 8E**).

To further evaluate systemic biosafety, serum biochemical analysis was performed. Serum levels of ALT, AST, UREA, and CREA in the LiH/MXene@MNs + NIR group remained within normal ranges, indicating no hepatotoxicity or nephrotoxicity (**Figure 8F-I**). In addition, H&E staining of vital organs (heart, liver, spleen, lung, kidney) at 1 and 3 weeks post-treatment showed no observable histopathological abnormalities, confirming excellent systemic biocompatibility (**Figure 8J and Figure S6**). Taken together, these findings demonstrate that LiH/MXene@MNs under NIR irradiation are not only effective in achieving rapid analgesia but also promote favorable tissue regeneration and maintain systemic safety, making them well suited for clinical translation.

## Conclusions

In summary, we developed a photothermal-activated MN system (LiH/MXene@MNs) enabling rapid, minimally invasive, and safe local anesthesia. Lidocaine hydrochloride was efficiently loaded into the needle tips, while MXene was confined to the backing layer. Under NIR irradiation, MXene in the backing layer generates localized heat within a safe temperature range within minutes, accelerating the diffusion of lidocaine through the microchannels formed by the MNs. This design enables a rapid onset within 5 min and sustained analgesia lasting up to 60 min via minimally invasive administration, comparable to conventional injections. Notably, the rapid analgesia achieved by LiH/MXene@MNs under NIR irradiation is sufficient to modulate peripheral and central neuronal activation, which contrasts with passive drug delivery patches. *In vitro* and *in vivo* evaluations confirmed that the system has strong mechanical strength, high drug-loading capacity, efficient skin penetration, and a favorable biosafety profile. LiH/MXene@MNs retains the advantages of conventional local anesthesia while avoiding its drawbacks and associated risks, and represents a promising alternative to traditional injected or topical local anesthesia.

## Methods

### Materials

PDMS molds and MXene were fabricated in the Polymer MN Laboratory at Beijing University of Chemical Technology 37. In this study, we used MXene, a representative two-dimensional titanium carbide composed of titanium (Ti) and carbon (C), belonging to the MXene family of transition metal carbides. Sucrose (99%) was purchased from Acros Organics (New Jersey, USA). PVA (Mw, 9-10 kDa) was obtained from Sigma-Aldrich (MO, USA). Lidocaine hydrochloride and rhodamine B were purchased from Aladdin (Shanghai, China). Cy3 was obtained from Duofluor Inc. (Wuhan, China). The Calcein-AM/PI Double Stain Kit (#40747ES80) was purchased from Yeasen Biotechnology Co., Ltd (Shanghai, China), and the Cell Counting Kit-8 (CCK-8, #BA00208) was obtained from Bioss (Beijing, China). The primary antibodies used in this study included c-Fos (#ab190289, Abcam), TRPV1 (#ab203103, Abcam), NeuN (#ab104224, Abcam), Iba1 (#ab178847, Abcam), and GFAP (#3670, Cell Signaling Technology). Fluorescently labeled secondary antibodies included Alexa Fluor 488 (#ab150077, Abcam) and Alexa Fluor 594 (#ab150116, Abcam). TUNEL cell apoptosis detection kit (G1504) was purchased from Wuhan Servicebio Technology Co., Ltd. (Wuhan, China). ELISA kits for rat TNF-α (E-EL-R2856), IL-6 (E-EL-R0015), and IL-1β (E-EL-R0012) were purchased from Elabscience Biotechnology Co., Ltd. (Wuhan, China).

### Preparation of LiH/MXene@MNs

The LiH/MXene@MNs comprised two main components: PVA MN tips loaded with LiH and a photothermal-responsive backing layer incorporating MXene. Briefly, a 25% (w/v) PVA solution was prepared by dissolving PVA in ultrapure water. LiH was then incorporated into the PVA solution at predetermined LiH:PVA mass ratios (0:4, 1:4, 1:2, 3:4, 1:1, and 2:1) to yield LiH/PVA mixtures for MN-tip formation. The backing layer formulation was prepared by dissolving PVA and sucrose in ultrapure water at a defined concentration ratio, followed by the addition of MXene at concentrations ranging from 0 to 200 μg·mL⁻¹. This backing layer provided the desired photothermal response under 808-nm NIR irradiation at specific power densities and MXene concentrations.

For MN fabrication, 50 μL of the 25% LiH/PVA solution was evenly dispensed onto the needle cavities of a custom PDMS mold. A vacuum was applied beneath the mold for 15 min to ensure complete filling of the cavities. Excess solution was removed, and residual traces on the surface were gently wiped away with a moistened tool. The mold was further vacuumed for 20 min to draw the LiH/PVA mixture fully into the needle tips and remove residual water, thereby preventing drug diffusion into the backing layer during subsequent processing. This vacuum process ensured that the LiH/PVA mixture was evenly distributed, minimizing the risk of uneven drug loading and diffusion into the backing layer. Second, 100 μL of the MXene/PVA solution was layered over the PDMS mold and vacuumed for 30 min to form the photothermal-responsive backing. The assembled MNs were dried overnight, demolded using a custom baseplate, and stored in a vacuum desiccator for subsequent characterization and application. The drying process was essential to ensure that both the needle tips and the backing layer were thoroughly solidified, preventing unintended mixing or migration of components during storage and application. The fabrication of blank MNs followed the same procedure without the addition of LiH or MXene. LiH@MNs were prepared without the addition of MXene, and MXene@MNs were prepared without the addition of LiH. Additionally, rhodamine B was incorporated into the solution in place of LiH to produce fluorescent MN patches, enabling visualization of drug distribution within the needle.

### Characterization of LiH/MXene@MNs

The MNs loaded with rhodamine B were observed and recorded using an optical microscope (SZX7, Olympus, Tokyo, Japan) to evaluate needle height and array integrity. The surface morphology and integrity of the MNs were further examined at higher resolution using scanning electron microscopy (SEM; JSM-7500F, JEOL, Japan). Elemental distribution was obtained via energy-dispersive X-ray spectroscopy to assess the distribution of LiH.

### Determination of drug loading

LiH standard solutions in PBS at various concentrations were prepared to establish a calibration curve. LiH/PVA MNs with different predetermined LiH:PVA mass ratios (without MXene) were immersed in PBS until completely dissolved. The LiH content in each sample was quantified using HPLC (Elite 230II, with a P 230II pump and UV 230II detector; Elite Analytical Instruments Co. Ltd., Dalian, China) at a detection wavelength of 254 nm, with a retention time of 12.6 min. The final drug loading of the fabricated MNs for each LiH:PVA mass ratio was calculated based on the calibration curve.

### Mechanical property test of LiH/MXene@MNs

To ensure adequate mechanical strength for skin penetration, LiH/PVA MNs with different mass ratios were evaluated to assess the balance between drug loading and mechanical performance. First, MNs were mounted on the displacement axis of a digital dynamometer (ESM301, Mark-10, USA), and force-displacement curves were recorded at a speed of 10 mm·min⁻¹. Next, under a predetermined load, a skin-mimicking sealing film was used to evaluate MN insertion, including the number of layers pierced and the insertion depth 11. Subsequently, excised porcine skin 38 was blotted to remove surface moisture, and rhodamine B-loaded MN patches were applied to assess the penetration rate; the skin was then sectioned to evaluate the penetration depth. Furthermore, optical coherence tomography (OCT; VivoSight Dx, Michelson Diagnostics Ltd., Kent, England) was employed to monitor the real-time penetration of MNs into rat skin. Finally, H&E staining was performed on dorsal rat skin sections to examine histological details 30.

### Photothermal performance evaluation of LiH/MXene@MNs

MNs loaded with varying concentrations of MXene (0, 50, 100, 150, 200 μg·mL⁻¹) were exposed to 808-nm laser irradiation at different power densities (0, 0.25, 0.5, 0.75, 1 W·cm⁻²) in rats *in vivo*. Real-time temperature changes were recorded using a thermal imaging camera (UTi260A, UNI-T Technology Co., Ltd., China). This setup enabled measurement of temperature distribution on the skin surface during NIR exposure, providing insight into the spatial uniformity of the photothermal effect. The correlation between photothermal conversion efficiency, MXene concentration, and laser power was analyzed. In addition, photothermal stability was evaluated through repeated on/off irradiation cycles *in vivo* 34.

### Influence of photothermal effects on drug release

To evaluate the photothermal effect on drug permeation, drug diffusion under different conditions was monitored using optical microscopy and a small animal *in vivo* imaging systems. Excised porcine skin, agarose gel, and intact rat skin were tested with MN patches under two settings: with NIR irradiation (NIR+) or without irradiation (NIR-). Optical microscopy was used to observe the diffusion of rhodamine B on excised porcine skin, agarose gel, and live rat skin, with measurements taken at 0 and 2 min. Additionally, the transdermal permeability of LiH was assessed using a Franz diffusion cell, with receiver fluid collected at different time points (0, 2.5, 5, 10, 15, 30, 45, 60 min) for LiH concentration analysis 39. For *in vivo* imaging of rat skin, Cy3-labeled MNs were used, and small animal *in vivo* imaging system were employed to track the drug permeation, with measurements taken at 0, 2, 5, 10, 15, 20, and 30 min 31. The study further analyzed the photothermal enhancement of drug diffusion by measuring cumulative release and radiant efficiency.

### Animal experiments

SPF male rats (6 weeks old; Beijing SPF Biotechnology Co., Ltd., Beijing, China) were maintained under standard husbandry conditions (40-60% relative humidity; 22 ± 2 °C) with unrestricted access to chow and water and a 12 h/12 h light-dark regimen. All animal procedures were reviewed and authorized by the ethics committee at Peking Union Medical College Hospital (Approval No.: XHDW-2024-126). The rats were divided into 8 groups (n = 6 per group): Control (non-incised); Surgery (PIM without treatment); LiH/Cream (post-surgery topical lidocaine cream); B-MNs (post-surgery blank MN patch, no drug); LiH@MNs (post-surgery lidocaine-loaded MN patch without NIR); MXene@MNs + NIR (MXene backing layer without lidocaine under NIR irradiation); LiH/MXene@MNs + NIR (post-surgery lidocaine-loaded, MXene-backed MN patch under NIR irradiation); and LiH-inj (post-surgery local lidocaine injection).

A plantar incision model was selected to evaluate the onset time and efficacy of local anesthesia following MN treatment based on changes in postoperative pain thresholds 40. Isoflurane anesthesia was delivered via inhalation and maintained throughout the procedure. The plantar surface was sterilized, after which a 1-cm lengthwise cut was created 0.5 cm distal to the proximal margin of the heel. The incision penetrated the skin, fascia, and flexor muscle, which was separated and longitudinally incised using blunt curved forceps, while preserving the origin and insertion points of the surrounding muscles. After hemostasis, the wound was sutured with 4-0 nylon thread, and a small amount of antibiotic ointment was applied. Rats in the control group underwent anesthesia, disinfection, and antibiotic treatment without any surgical intervention. Following surgery, rats were returned to their original cages. After 36 h, the corresponding treatments were administered to each group.

### Pain behavioral test

Pain behavior tests were conducted at specific time points, including the day before surgery and postoperative time points (baseline, 5, 10, 20, 30, 45, 60, 90, and 120 min after treatment), to evaluate the onset and duration of anesthesia under different treatments. Prior to each test phase, rats were allowed to adapt to the testing environment for 30 min. The 50% PWT was determined using the up-and-down method. A series of von Frey filaments (Bioseb, USA; 1.0-15.0 g: 1.0, 1.4, 2.0, 4.0, 6.0, 8.0, 10.0, and 15.0 g) was used to stimulate the incision site on the plantar surface, and withdrawal responses were recorded. If a rapid paw withdrawal was observed, it was marked as a positive response (X); no withdrawal was marked as negative (O). This process continued until the sixth stimulus after the first observed response change. The calculation formula for the 50% PWT is as follows: 50% PWT = 10 ^(Xf + kδ)^ ·10^-4^, where Xf is the final von Frey filament value (expressed in logarithmic units), k is the value for the positive/negative response, and δ is the average increment between von Frey filaments (expressed in logarithmic units; δ = 0.231) 41.

Additionally, the Hargreaves test was employed to quantify plantar thermal sensitivity with a movable radiant heat source (SH-1000 Plantar Test, Wokesi Bio-Tech Co., Ltd., Shanghai, China). During the test, rats were placed in a 79 cm × 40 cm × 6.5 cm testing platform, and an infrared emitter was used to deliver an invisible infrared light source (light intensity: 10%) to the plantar surface of the rat's foot. When the rat exhibited a paw withdrawal response, the displacement was recorded, and the PWL was determined. The cutoff time was typically set at 30 s to prevent tissue damage.

### Immunofluorescence staining

Rats were anesthetized with isoflurane 36 h after surgery and perfused sequentially with 20 mL of normal saline, followed by 15 mL of 4% paraformaldehyde 29. The L4-L5 DRGs and the lumbosacral enlargement of the spinal cord were collected. Tissues were post-fixed in 4% paraformaldehyde for 24 h and cryoprotected in 30% (w/v) sucrose. After freezing, cryosections were prepared at 16 μm for spinal cord and 8 μm for DRG. c-Fos and TRPV1 staining in the DRG reflected the activation of TRPV1-positive neurons, while in the spinal cord, c-Fos and NeuN were used to label neuronal activation. Additionally, Iba-1 and GFAP were used to label microglia and astrocytes, respectively. The number of positive neurons and the MFI were calculated using ImageJ software.

### Wound healing assessment

Following the methods described above, behavioral tests were conducted on day 7 post-surgery to assess pain recovery. Subsequently, animals were sacrificed and periwound tissue around the plantar incision was excised for fixation in 4% paraformaldehyde. Specimens were paraffin-embedded and cut into sections, followed by H&E and Masson's trichrome staining. CVF was quantified with ImageJ.

### Biosafety of LiH/MXene@MNs

*In vitro* cytotoxicity assays were performed on rat BMSCs (CP-R131, Procell Life Science & Technology Co., Ltd., China) and mouse NIH/3T3 cells (CL-0171, Procell Life Science & Technology Co., Ltd., China) 30, 31. The cells were cultured in DMEM supplemented with 10% FBS, penicillin (100 U·mL⁻¹), and streptomycin (100 U·mL⁻¹). The cultures were maintained in a humidified incubator at 37 °C with 5% CO_2_. Cell viability and cytotoxicity after 24 h of treatment with different concentrations of MNs (25, 50, 100, 200, 400, and 800 μg·mL⁻¹) were evaluated using the Calcein-AM/PI Double Stain Kit and the CCK-8 assay.

To ensure the safe application of MNs, the recovery of puncture sites after MN treatment and the effects of photothermal treatment on the skin were evaluated. Specifically, MNs loaded with rhodamine B were inserted into the depilated dorsal skin of rats, and the resulting puncture marks were observed at different time points (0, 30, 60, and 120 min). To assess the safety of the thermal effect, MNs were applied to the dorsal skin of rats, and after near-infrared stimulation, they were irradiated at different temperatures (40, 50, 60, and 70 °C) for 2 min or 5 min. Three hours later, the corresponding areas on the rats' backs were examined for signs of inflammation, redness, or swelling. In addition, to further evaluate the local biosafety of MN patches under NIR activation, an independent cohort of rats received MN application to the plantar skin. Under the same NIR irradiation protocol described above, the local skin surface temperature was controlled at 40 °C or 50 °C for 2 min to match the irradiation duration used in the behavioral experiments. On day 3 post-treatment, plantar skin tissues at the application site were collected for histological and inflammatory assessments. Briefly, samples were fixed in 4% paraformaldehyde, paraffin-embedded, and sectioned, followed by H&E staining to examine tissue morphology. Apoptosis was evaluated using a TUNEL assay kit. In parallel, additional tissues from the same region were homogenized, and the protein levels of TNF-α, IL-6, and IL-1β were quantified using ELISA kits.

For the hemolysis assay, blood was obtained via orbital sinus sampling in rats. The LiH@MNs, MXene@MNs, and LiH/MXene@MNs were dissolved in 0.9% saline to prepare solutions at different concentrations (25, 50, 100, 200, 400, and 800 μg·mL⁻¹) 42. PBS and 1% Triton X-100 were used as the negative and positive controls, respectively. The MN solution was mixed with a 5% red blood cell suspension (1:1, v/v) and incubated at 37 °C for 1 h. The absorbance (OD) of the mixture was measured at 540 nm using a microplate reader. The hemolysis rate was calculated using the following formula:



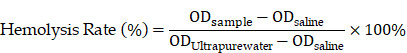



Serum was separated by centrifugation and used for biochemical measurements of ALT, AST, UREA, and CREA as indicators of hepatic and renal function. At 1 and 3 weeks after treatment, H&E staining was performed on the heart, liver, spleen, lung, and kidney tissues of rats to assess tissue damage or inflammatory responses.

### Statistical analysis

Data are presented as the mean ± standard error (SE). Statistical testing was carried out in GraphPad Prism 9 (GraphPad Software, USA). Comparisons between two groups employed an unpaired, two-sided t test, whereas datasets with more than two groups were evaluated by one-way ANOVA with Tukey's multiple-comparisons procedure. Unless stated otherwise, *P* < 0.05 indicated significance.

## Supplementary Material

Supplementary figures.

## Data Availability

The data that support the findings of this study are available from the corresponding author upon reasonable request.

## Abbreviations

MNs: microneedles
NIR: near-infrared
LiH: lidocaine hydrochloride
PVA: polyvinyl alcohol
PDMS: polydimethylsiloxane
SEM: scanning electron microscopy
EDS: energy-dispersive X-ray spectroscopy
HPLC: high-performance liquid chromatography
BMSCs: bone marrow derived mesenchymal stem cells
PIM: plantar incision model
PWT: paw withdrawal threshold
PWL: paw withdrawal latency
DRG: dorsal root ganglion
SG: substantia gelatinosa
TRPV1: transient receptor potential vanilloid 1
MFI: mean fluorescence intensity
NeuN: neuronal nuclei
Iba-1: ionized calcium binding adaptor molecule-1
GFAP: glial fibrillary acidic protein
CVF: collagen volume fraction
SE: standard error
