# Supplementary material for: Near-infrared light-activated lidocaine microneedle patch for rapid local anesthesia
Source: Theranostics. 2026 Mar 9;16(10):5259–77. doi: 10.7150/thno.129132 (PMC13080483; doi:10.7150/thno.129132)

Supplementary file

Near-infrared light-activated lidocaine microneedle patch for rapid local anesthesia

Shuailei Wang[1#]; Ze Qiang Zhao[2, 3#]; Yumiao He[1]; Bo Zhi Chen[2, 3]; Hongju Liu[1]; Xin Dong Guo[2, 3*]; Yuguang Huang[1*]

[1]Department of Anesthesiology, Peking Union Medical College Hospital, Chinese Academy of Medical Sciences & Peking Union Medical College, Beijing, 100730, China

[2]State Key Laboratory of Organic-Inorganic Composites, Beijing University of Chemical Technology, Beijing 100029, China.

[3]Beijing Laboratory of Biomedical Materials, College of Materials Science and Engineering, Beijing University of Chemical Technology, Beijing 100029, China

Figure S1. SEM image of the LiH/MXene@MNs and elemental mapping of a single MN by EDS. Red represents carbon (C) and purple represents titanium (Ti). The Ti is the representative element of MXene.

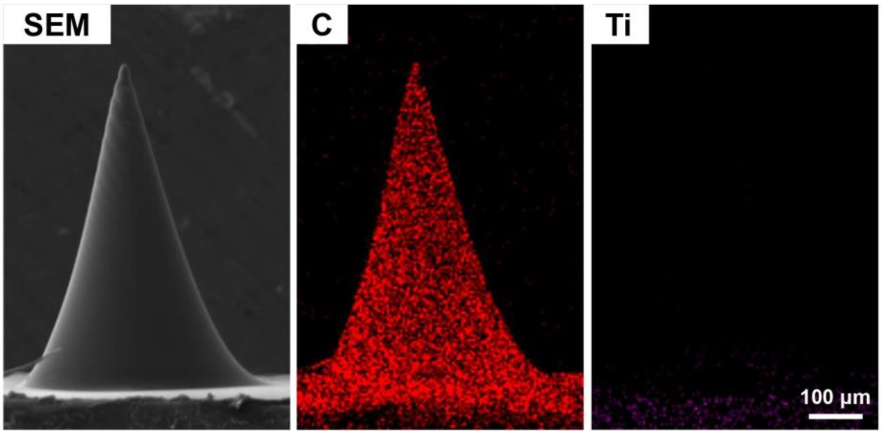

Figure S2. Histological evidence of MNs insertion into rat plantar skin (H&E staining). Scale bar: 100 μm.

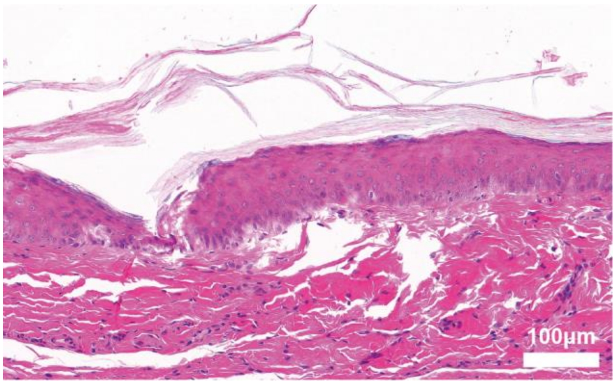

Figure S3. The hemolysis rate of red blood cells incubated with PBS (negative control), and 1% Triton X-100 (positive control). Scale bar: 50 μm.

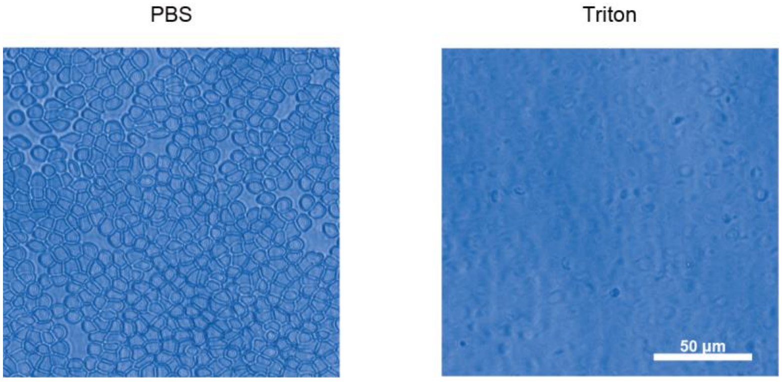

Figure S4. Temperature changes in the rat's paw following application of LiH/MXene@MNs and 2 minutes of NIR treatment (N=6).

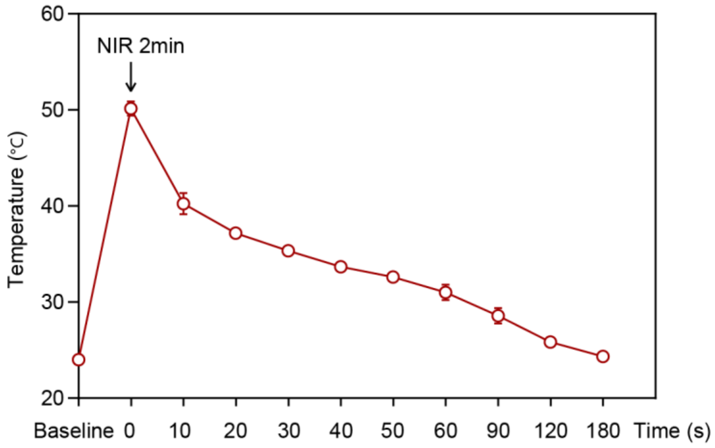

Figure S5. Immunofluorescence staining of Iba-1 and GFAP at 1 h post-treatment. (A) Representative immunofluorescence images of Iba-1 (green) and GFAP (red) staining in L4-5 DRG. (B) MFI of Iba-1 at different groups. (C) MFI of GFAP at different groups. All data are shown as mean ± SE (n = 6 slides/group from 6 rats) and analyzed by one-way ANOVA (ns: not significant and $^{**}p < 0.01$). All scale bars:100 μm.

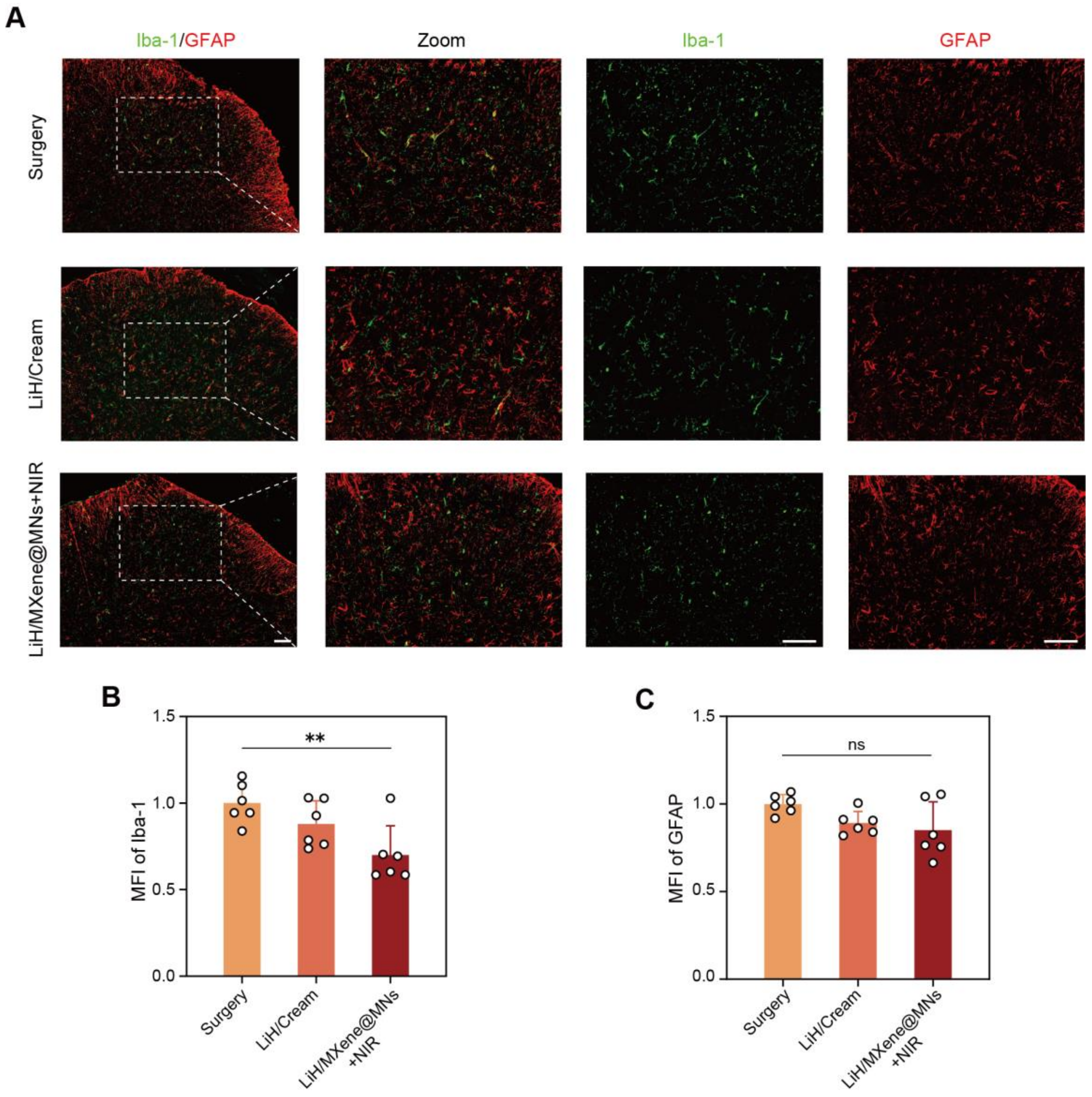

Figure S6. H&E staining of heart, liver, spleen, lung, kidney in rats in different groups at 3 weeks after treatment. Scale bars: 500 μm.

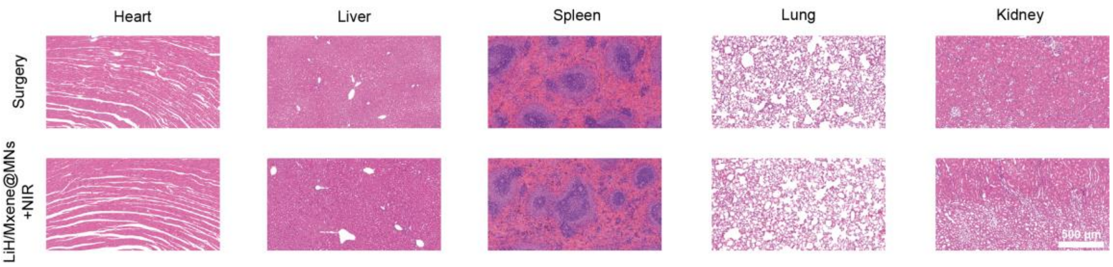

Supplement: Supplementary file 1 — Supplementary figures. [file thnov16p5259s1.pdf]
